# Birth Weight and Incidence of Surgical Obstetric Brachial Plexus Injury

**Published:** 2015-04-28

**Authors:** Rahul K. Nath, Meera B. Avila, Sonya E. Melcher, Devin K. Nath, Mitchell G. Eichhorn, Chandra Somasundaram

**Affiliations:** Texas Nerve and Paralysis Institute, Houston, TX

**Keywords:** macrosomia, obstetric brachial plexus injury (OBPI), shoulder dystocia, Mod Quad surgery, birth weight

## Abstract

**Objectives:** (1) To analyze the birth weight of obstetric brachial plexus injury (OBPI) patients requiring one or more reconstructive surgeries and (2) to analyze whether there is any difference in the severity of the injury, and the outcome of the surgery between the macrosomic and nonmacrosomic OBPI patients. **Study Design:** An observational cohort study was performed on 100 consecutive patients treated with surgery at the Texas Nerve and Paralysis Institute. Ninety of the 100 patients underwent the modified Quad surgery, which improves the shoulder abduction and overall shoulder function. All OBPI patients in our study were assessed preoperatively and postoperatively by evaluating video recordings of active shoulder abduction. **Results:** *Using a 4000 g definition of macrosomia, 52% of patients would be considered macrosomic, and using a 4500 g definition of macrosomia, 18% of patients are considered macrosomic in our study.* Permanent injury occurs also in average-birth-weight children. **Conclusions:** *A significant percentage (48%-82% depending on definition of macrosomia) of OBPI patients requiring major reconstructive surgery had birth weights which would put them in the “normal” birth weight category.* In addition, we found that there was no significant difference in the severity of the injury, and the outcome of the modified Quad surgical procedure between macrosomic and nonmacrosomic OBPI patients. However, there was a significant improvement in shoulder movement in both macrosomic and nonmacrosomic patients after modified Quad surgery.

Up to 5000 newborns are affected by obstetric brachial plexus injury (OBPI) annually in the United States.^1^ Obstetric brachial plexus injury occurs at a rate of 0.1% to 0.6% of live births.^2^^,^^3^ Many of these injuries are transient; however, most of the OBPI patients never recover full function and develop permanent injuries.^4^^-^6 The obstetric community has been unable to decrease the frequency of OBPI for more than 80 years.^7^ The severity of injury to the brachial plexus, which is the most complex peripheral neural unit, can range from neurapraxia (stretch) to neurotmesis (rupture) or spinal cord avulsion.^8^ Risk factors associated with OBPI include gestational diabetes mellitus, vacuum extraction, use of forceps, shoulder dystocia, and macrosomia.^9^^-^11 It has also been reported that the twisting and the extension of the fetal head, which will increase the stretching of the neck, might cause OBPI.^12^

Macrosomia, which occurs in almost 10% of all births, has been defined as both greater than 4000 g^13^ and greater than 4500 g.^9^^,^^14^^-^16 The chance of a fetus being macrosomic can be increased by prolonged pregnancy^10^ or maternal diabetes.^17^ The Royal College of Obstetricians and Gynaecologists reported that OBPI is a major complication associated with fetal macrosomia and is one of the most significant complications of shoulder dystocia.^14^ In our study of 100 consecutive OBPI patients requiring reconstructive surgery, we report that a significant percentage of these OBPI patients are nonmacrosomic.

## METHODS AND CLINICAL MATERIAL

An observational cohort study was performed to analyze the average birth weight of 100 consecutive OBPI patients surgically treated in 2007 at the Texas Nerve and Paralysis Institute. All patients in the study have had at least 1 reconstructive surgery related to the initial nerve injury. The indication for surgery is same for macrosomic and nonmacrosomic patients. However, most of them (90%) have undergone the modified Quad surgical procedure, 18 which is a modification of the combination of muscles released at their insert positions to improve upon a previously described operation.^19^ Teres minor was then mobilized and the arm placed in full abduction and external rotation. An incision was made into the teres minor and the tendons of latissimus dorsi and teres major were individually sutured into this. The wound was closed in 2 layers in absorbable suture over a drain.

**Figure 1:**
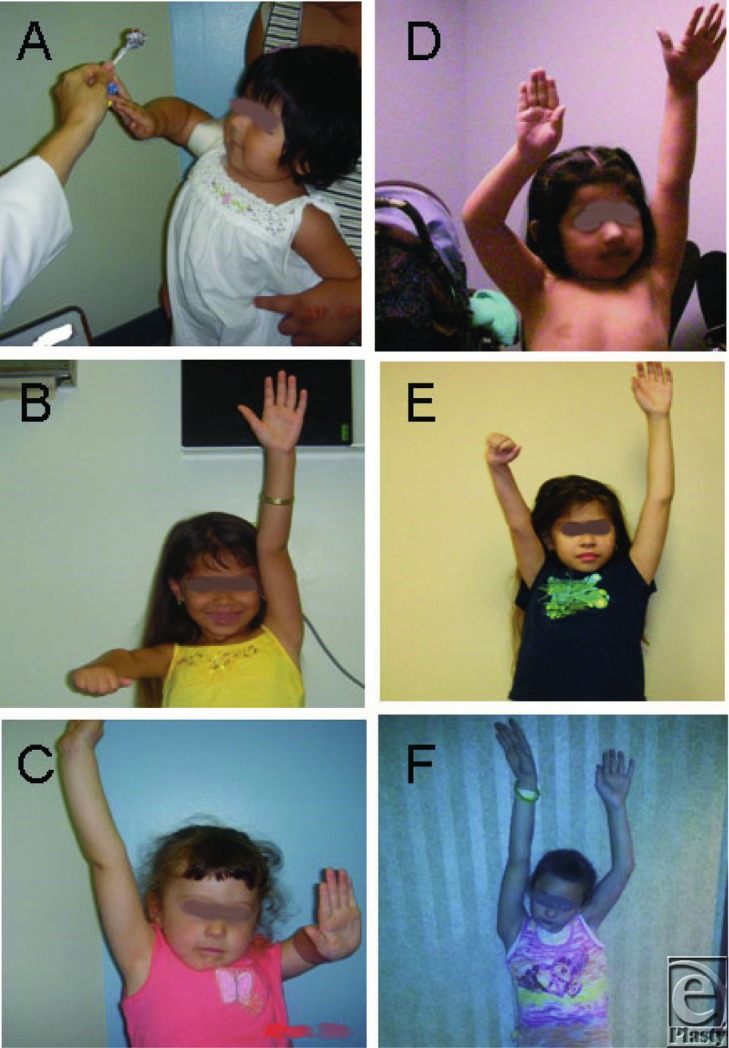
Comparisons of pre- and postoperative shoulder abduction in OBPI patients. Preoperative photograph of a macrosomic patient (a), and nonmacrosomic patients (b) and (c), demonstrating limitation of shoulder movement; same patients at least 1 year after modified Quad surgery with almost normal shoulder abduction (d, e, and f).

Postoperative care included immobilization in an abduction “Statue of Liberty” splint for 4 weeks after which the child was placed in the splint at nighttime only for a further 6 weeks. Full shoulder movement was permitted after the initial 4-week postoperative period. Rapid improvement in shoulder abduction was noted in all patients. Physiotherapy was prescribed 3 times a week for at least 3 months, and swimming was encouraged.^18^ All surgeries were performed by the same surgeon (R.K.N.), whose practice has focused on reconstructive surgery in this population for the past 19 years (Fig 1).

The following data points were obtained and recorded: birth weight, instruments required for delivery, surgeries received, movement at birth, and history of shoulder dystocia. The average birth weight of all patients was collected, as well as the average birth weight of patients grouped by the presence or lack of dystocia and movement or no movement at birth. The active shoulder abduction in all patients was assessed preoperatively and postoperatively.^20^

### Statistical Analysis

The Student *t* tests were conducted using Microsoft Excel 2003 with the Analyze-It plug-in (Redmond, Wash, and Leeds, United Kingdom) to determine the statistical differences between pre- and postoperative shoulder abduction, and compared between macrosomic and nonmacrosomic patients. The *P* values were 2-tailed and considered significant if ≤0.05.

## RESULTS

Females composed 51% of the patients analyzed and males composed 49%. The mean average birth weight of all patients analyzed in the study was 4029 g. The minimum birth weight was 2126 g, and the maximum birth weight was 5046 g. The percentage of patients weighing greater than 4000 g was 52%, while the percentage of patients weighing less than 4000 g was 48%. The percentage of patients weighing greater than 4500 g was 18%, while the percentage of patients weighing less than 4500 g was 82%. The rate of documented shoulder dystocia among children with permanent brachial plexus injury was 96%. Birth weight was significantly higher in patients with documented shoulder dystocia (4063 ± 733 g) than in those without documented shoulder dystocia (3210 ± 490 g).

Instruments were used in the deliveries of 39% of the patients studied, including the use of a vacuum and/or forceps. Of the 100 patients, 71% experienced no movement of the affected arm at birth, while 29% of patients experienced movement only in their fingers. The mean birth weight in instrument-assisted deliveries was not significantly different from that in spontaneous deliveries. In addition, mean birth weight among patients with no finger movement was (4022 ± 542 g) also not significantly different from that among the patients with finger movement at birth (4048 ± 490 g).

The following surgeries were performed on these patients: modified Quad surgery, triangle tilt surgery, nerve grafting, posterior glenohumeral capsulorrhaphy, biceps tendon lengthening, humeral osteotomy, and anterior capsule release.

The severity of the injury based on the shoulder movement preoperatively was not significantly different between the macrosomic (3.3 ± 0.9, 93 ± 46°) and nonmacrosomic (2.9 ± 0.8, 70 ± 40°) OBPI patients (Table 1).

Since most of the patients in this study have had the modified Quad surgery, we considered the outcome of this surgery to determine whether there is any difference in the improvement between the macrosomic and nonmacrosomic patients. There was no significant difference in the surgical outcome between these 2 groups of patients (Table 2), although there was highly significant improvement following this surgery in all the patients, in both macrosomic and nonmacrosomic patients (Table 3). We did not have complete pre- or postmod Quad data for 22 patients, and those patients were excluded for calculating shoulder abduction and the angle (Table 1-3).

## DISCUSSION AND CONCLUSIONS

Our results confirm the fact that a significant percentage of patients (52%) are macrosomic (by the >4000 g definition). However, our results also revealed that a significant percentage of OBPI patients (48%) would not be considered macrosomic. Our findings indicate that nonmacrosomic fetuses frequently experience shoulder dystocia and develop permanent OBPI, despite the fact that macrosomia is said to be one of the primary indicators of permanent OBPI.^21^ Alsammani and Ahmed^22^ recently reported only 0.96% (4 among 418) of macrosomic patients (mean fetal birth weight, 4.59 ± 0.56 kg) were encountered with brachial plexus birth palsy. Mollberg et al^21^ reported that the risk of an infant developing OBPI increases sharply beyond the 4500-g-mark. In the study by Mollberg et al, the definition of permanency of injury was not clearly stated. In addition, the lack of evaluation by specialists experienced in managing the long-term functional aspects of these injuries is a probable reason for the underreporting of the frequency of permanent functional deficits in the OBPI population. The incidence of permanent injury is significantly higher in reports written by specialists having interaction with these patients later in life, when permanency and severity of injury can be more accurately determined.^4^^-^6

Gurewitsch et al^23^ reported that more than 90% of permanent brachial plexus palsy patients were associated with shoulder dystocia. These data are consistent with our study report; that is, 96% of our OBPI patients are also with shoulder dystocia. In our study, patients were all also permanent brachial plexus injury patients, who required major reconstructive surgery. This study also demonstrates a near universal association of shoulder dystocia with the most severe brachial plexus injuries and substantiates the need to investigate and manage shoulder dystocia to prevent these injuries.

In addition, it is important that delivering caregivers consider that nonmacrosomic babies may also sustain injury to OBPI, because of other causes such as twisting and the extension of the fetal head, which will increase the stretching of the neck.^12^

### Conclusions

A significant percentage (48%-82% depending on the definition of macrosomia) of OBPI patients requiring major reconstructive surgery had birth weights, which would put them in the “normal” birth weight category. In addition, we found that there was no significant difference in the severity of the injury, and the outcome of the modified Quad surgical procedure between macrosomic and nonmacrosomic OBPI patients.

## Figures and Tables

**Table 1:** Shoulder abduction preoperatively in macrosomic and nonmacrosomic patients*

	Nonmacrosomic	Macrosomic	*P*
Shoulder abduction	2.9 ± 0.8	3.3 ± 0.9	.22
Abduction angle	70 ± 40°	93 ± 46°	.08
	n = 56	n = 12	

*Shoulder abduction angle <30° = abduction in Mallet function 1- 2, abduction is not possible; angle 30-90° equal 3, abduction is difficult; >90° equals 4, abduction is easy; 180° angle equals 5, abduction is normal.

**Table 2:** Outcome of modified Quad surgery between macrosomic and nonmacrosomic OBPI patients*

	Shoulder abduction	Abduction°	*P*
Nonmacrosomic OBPI patients	1.4	79°	0.12
		(n = 56)	
Macrosomic OBPI patients	1.3	58°	0.35
		(n = 12)	

*Shoulder abduction angle <30° = abduction in Mallet function 1- 2, abduction is not possible; angle 30-90° equal 3, abduction is difficult; >90° equals 4, abduction is easy; 180° angle equals 5, abduction is normal.

**Table 3:** Compared the pre- and postoperative shoulder abduction, and the abduction angle in macrosomic and nonmacrosomic patients*

	Premod Quad		Postmod Quad	
	Shoulder abduction†	Abduction angle†	Shoulder abduction†	Abduction angle†
Nonmacrosomic	2.9 ± 0.8	70 ± 40°	4.3 ± 0.5	149 ±22°
Macrosomic	3.3 ± 0.9	93 ± 46°	4.6 ± 0.5	151 ±30°

*Mean ± STD, *P* < .00001. †n= 56 (nonmacrosomic patients). n= 12 (macrosomic patients). Shoulder abduction angle <30° = abduction in Mallet function 1- 2, abduction is not possible; angle 30-90° equal 3, abduction is difficult; >90° equals 4, abduction is easy; 180° angle equals 5, abduction is normal.
